# Supplementary figures and images for: DSS-induced inflammation in the colon drives a proinflammatory signature in the brain that is ameliorated by prophylactic treatment with the S100A9 inhibitor paquinimod
Source: J Neuroinflammation. 2021 Nov 10;18:263. doi: 10.1186/s12974-021-02317-6 (PMC8578918; doi:10.1186/s12974-021-02317-6)

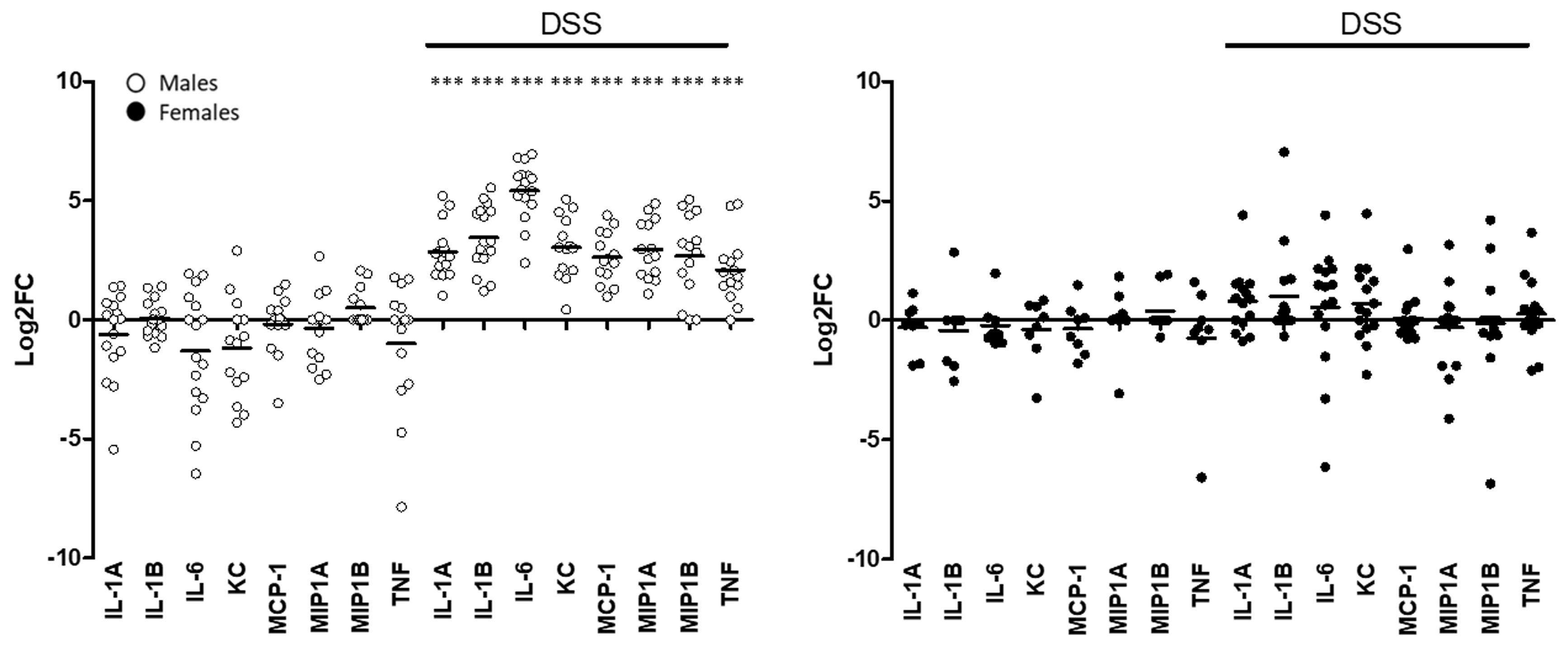

Supplement: Supplementary file 1 — Additional file 1. Inflammatory cytokines/chemokines detected in male mice treated with DSS. Mice were treated with 2% DSS for 7 days. Colons were isolated, homogenized and levels of the indicated inflammatory cytokines/chemokines were measured by CBA. The full CBA dataset for male (white) and female (black) is presented, where each individual dot depicts the log2FC of cytokine/chemokine expression in one mouse. Student’s t test comparing DSS to control for each cytokine/chemokine. *** = p < 0.0005. [file 12974_2021_2317_MOESM1_ESM.tif]

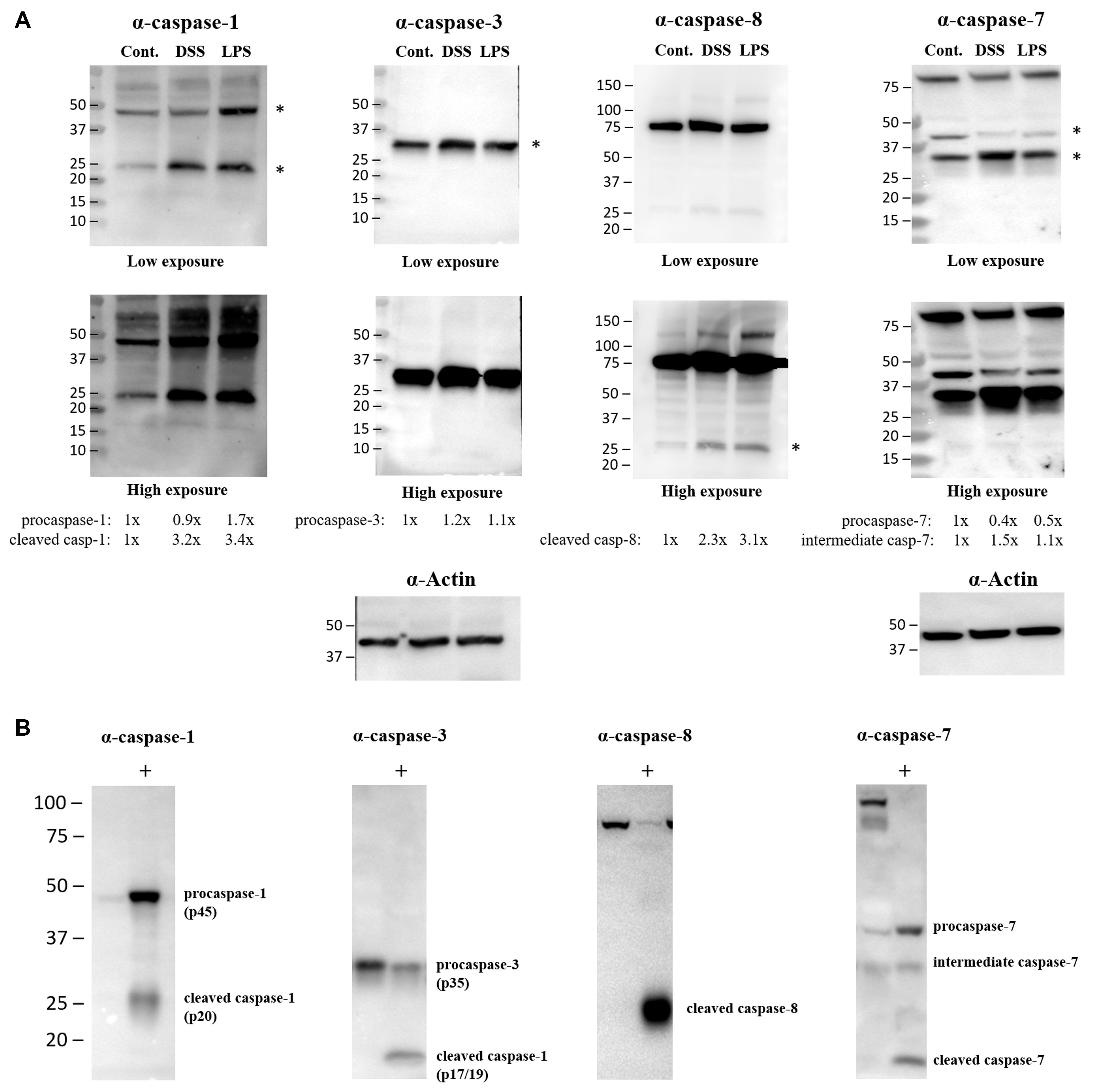

Supplement: Supplementary file 2 — Additional file 2. Caspase expression in the brain. Lysates from digested brains isolated from control, DSS or LPS-treated mice were stained with antibodies against caspase-1 p20 (shown in Fig. 2C), caspase-3, caspase-8, caspase-7 or actin (A). Bands labeled with an asterisk were quantified (A). Mouse splenocytes treated with 10 μM staurosporine in vitro were used as a positive control (+ lane) to measure cleaved caspases (B). [file 12974_2021_2317_MOESM2_ESM.tif]

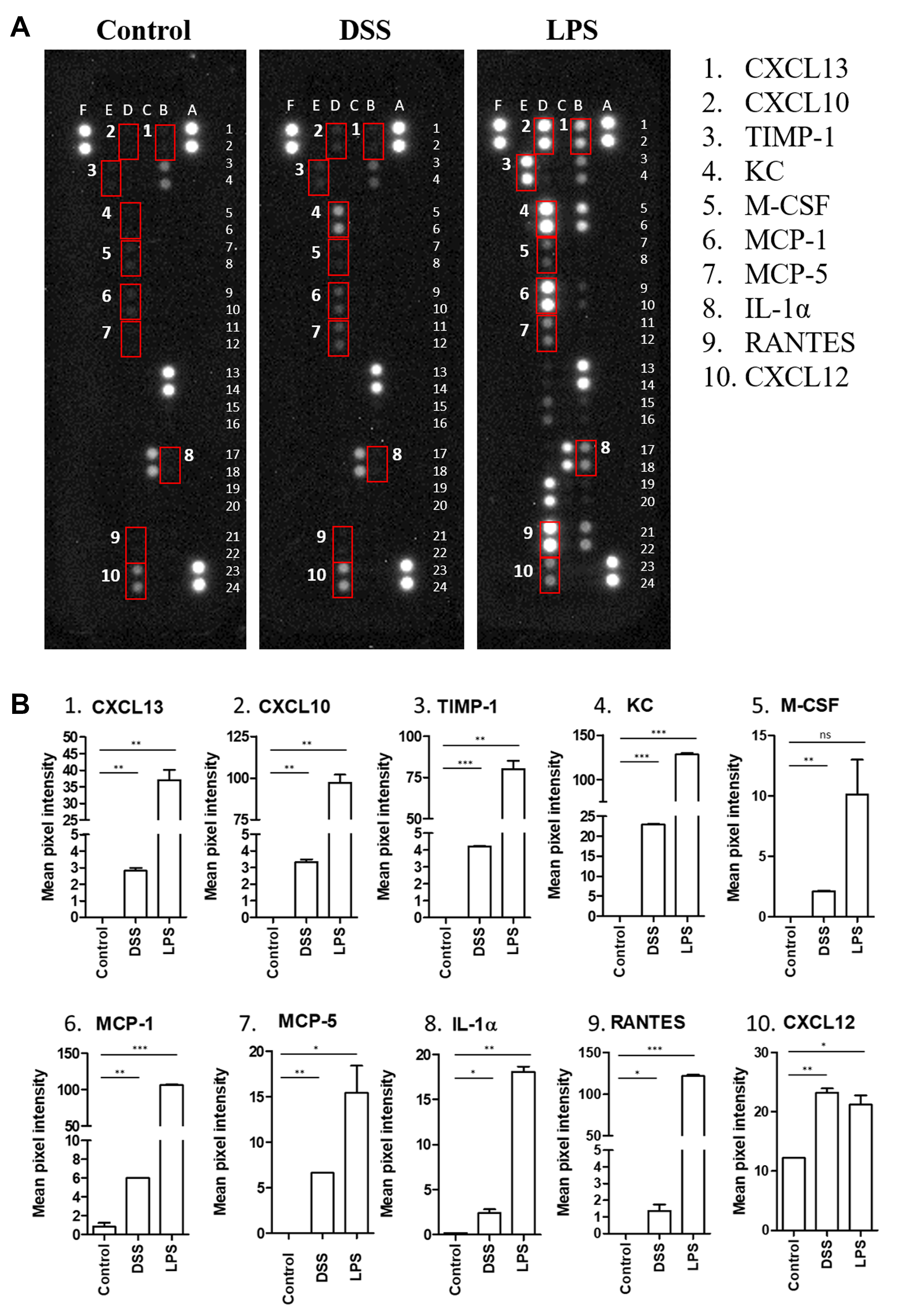

Supplement: Supplementary file 3 — Additional file 3.Cytokine/chemokine levels in DSS and LPS-treated brains. Male mice were treated with 2% DSS for 7 days or injected with 100 μg LPS for 24 h. Brain tissue was collected from control, DSS-treated and LPS-injected mice and ~ 10,000 μg whole brain homogenates were incubated with the capture antibody mixture and applied to membranes spotted with detection antibodies for 40 inflammatory cytokines/chemokines (in duplicate). The representative blots (1 control, 1 DSS and 1 LPS) shown in Fig. 2 are overexposed to better visualize qualitative changes in expression, and the red boxes indicate the 10 cytokines/chemokines that were significantly upregulated in the brain isolated from the DSS-treated animal (A). Mean pixel intensity was quantified for each spot using ImageJ, and expressed relative to control (B). Student’s t test comparing DSS or LPS to control. *** = p < 0.0005, ** = p < 0.005, * = p < 0.05. [file 12974_2021_2317_MOESM3_ESM.tif]

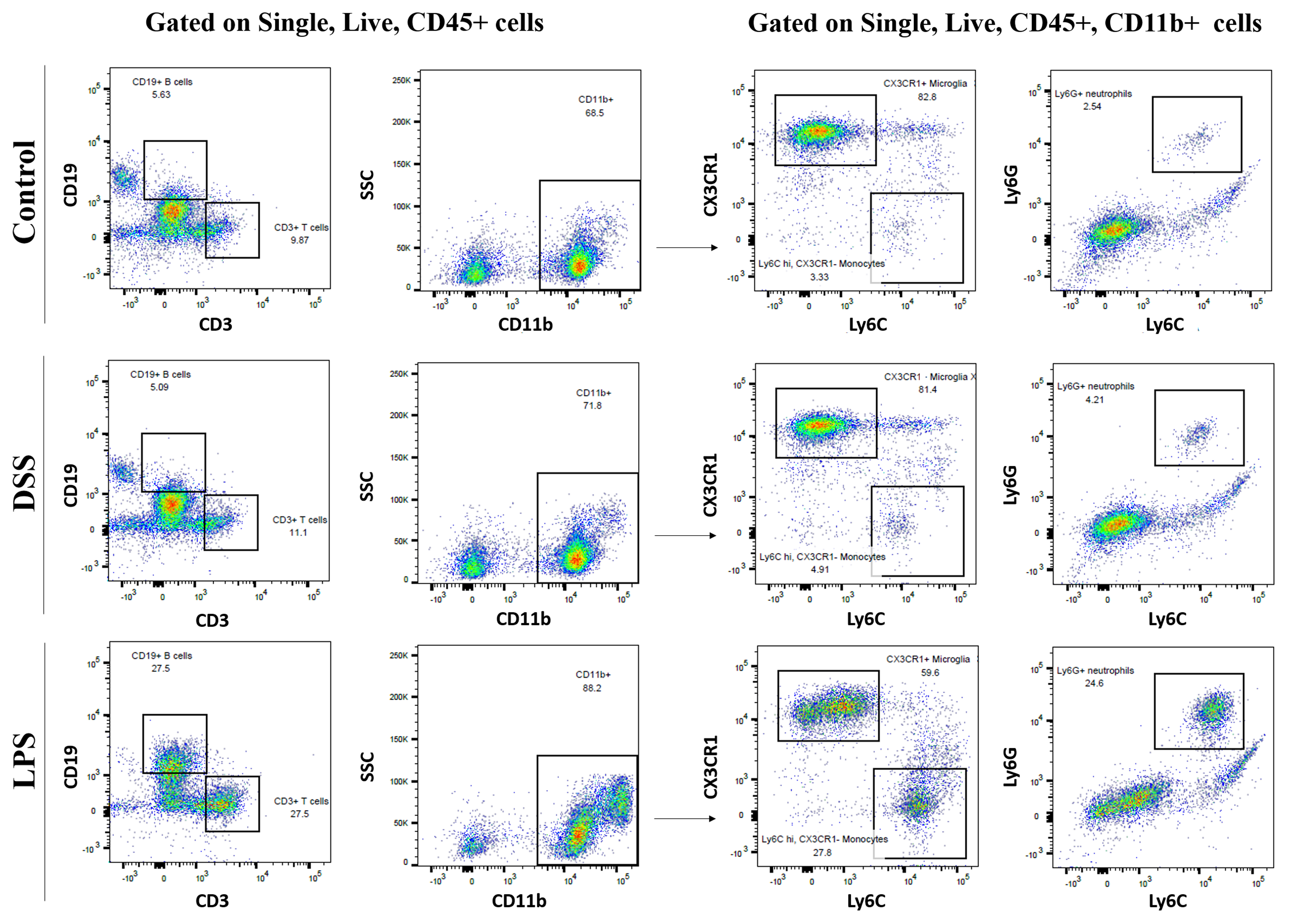

Supplement: Supplementary file 4 — Additional file 4. Flow cytometric analysis of immune cell populations in the brain in mice with colitis. Male mice were treated with 2% DSS for 7 days or injected with 100 μg LPS for 24 h. Brain tissue was collected from control, DSS-treated and LPS-injected mice. Brain tissue was digested and isolated cells were stained for the indicated markers. Single, live, CD45 + cells were gated and CD11b, CD3 and CD19 expression was assessed. Within the CD11b-positive population, CX3CR1, Ly6C and Ly6G expression were assessed. Representative flow plots are shown from a control, DSS and LPS brain. Data are representative of 3 independent experiments. Results were analyzed using FlowJo software. [file 12974_2021_2317_MOESM4_ESM.tif]

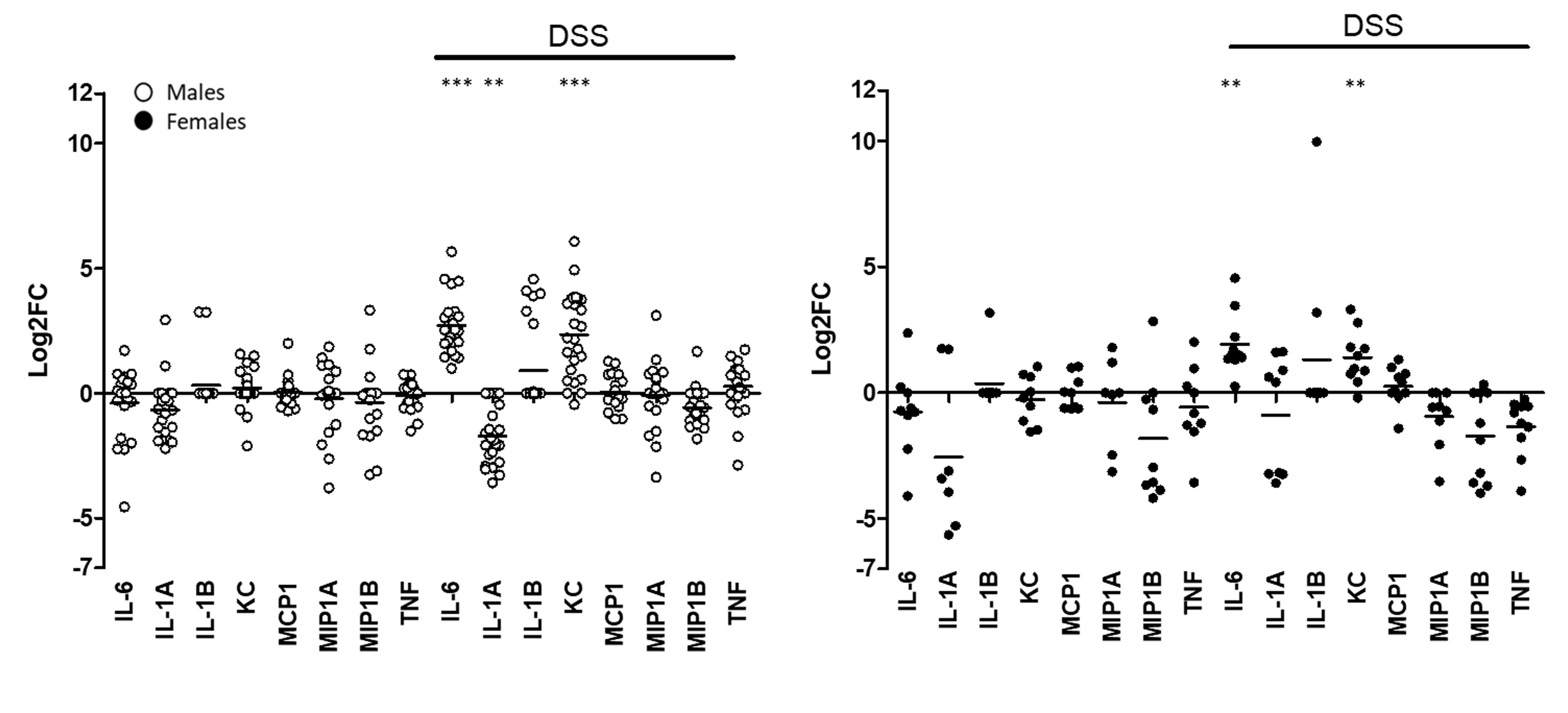

Supplement: Supplementary file 5 — Additional file 5. Changes in inflammatory cytokines in serum from DSS-treated males and females. Blood was collected and CBA was performed on the serum from control and DSS-treated mice. Dataset depicts the log2FC in cytokine/chemokine expression from male (white) and female (black) mice. Student’s t test comparing DSS to control for each cytokine/chemokine indicated on the x-axis. *** = p < 0.0001, ** = p < 0.005. [file 12974_2021_2317_MOESM5_ESM.tif]
